# Accuracy of Labial Salivary Gland Biopsy in Suspected Cases of Sjogren’s Syndrome

**DOI:** 10.7759/cureus.74746

**Published:** 2024-11-29

**Authors:** Abdulrahman I AlMannai, Khadija Alaradi, Sayed Ali I Almahari

**Affiliations:** 1 Otolaryngology - Head and Neck Surgery, Salmaniya Medical Complex, Manama, BHR; 2 Pathology and Laboratory Medicine, Salmaniya Medical Complex, Manama, BHR

**Keywords:** autoimmune disease, clinical correlation, diagnostic methods, labial biopsy, salivary gland, sicca, sjögren’s syndrome

## Abstract

Introduction: Sjögren’s syndrome (SS) is a chronic autoimmune disorder primarily characterized by dysfunction of the exocrine glands, leading to dryness of the eyes and mouth (sicca symptoms). Labial salivary gland biopsy (LSGB) is a key diagnostic tool used to confirm SS through histopathological analysis. LSGB evaluates lymphocytic infiltration in the salivary glands, a hallmark of SS. Despite its utility, discrepancies between LSGB results and other diagnostic methods, like serology and clinical examination, persist. Given the potential for false negatives, especially in early-stage or mild diseases, LSGB is often used alongside other diagnostic tools. Assessing its diagnostic accuracy and correlation with clinical, demographic, and serological factors is critical for improving diagnostic precision in SS.

Objectives: This study aims to evaluate the diagnostic accuracy of LSGB in suspected SS cases, focusing on its correlation with clinical diagnoses, serological markers, and demographic factors. It also investigates whether LSGB can serve as a standalone diagnostic tool or should be integrated with other methods to enhance accuracy.

Methods: This retrospective study evaluated 166 patients who underwent LSGB for suspected SS. Results were classified as "suggestive" or "not suggestive" of SS based on the histopathological evidence of lymphocytic infiltration. The relationship between LSGB outcomes and various demographics, serological markers, and the presence of other autoimmune diseases was examined. Statistical analyses assessed the significance of these associations. Categorical data were presented by frequency with percentage, and continuous data by mean with standard deviation (SD). Analyses were conducted using the Statistical Package for the Social Sciences (SPSS) version 27.0, with a P-value < 0.05 considered statistically significant.

Results: Among the 166 patients, 55 (33.1%) had LSGB results suggestive of SS, while 111 (66.9%) had non-suggestive findings. A significant association was found between antinuclear antibody (ANA) positivity and suggestive LSGB results (P = 0.003), indicating that ANA-positive patients were more likely to show histopathological evidence of SS. No significant associations were found with other serological markers, except for a near-significant trend with anti-Ro (anti-Ro/SSA antibodies) (P = 0.062). Age did not significantly influence biopsy outcomes (P = 0.580). The presence of other autoimmune diseases was significantly associated with suggestive LSGB findings (P = 0.034).

Conclusion: LSGB remains a valuable diagnostic tool for SS, especially when serological and clinical findings are inconclusive. The study confirmed significant associations between ANA positivity and suggestive LSGB results, as well as the influence of other autoimmune diseases on histopathological outcomes. While Schirmer's test may detect ocular dryness, its correlation with LSGB findings was limited. LSGB should not be used as a standalone diagnostic measure but integrated with other tools, including serology and imaging, to improve accuracy. Future research should explore combining LSGB with salivary gland ultrasonography (SGUS) to enhance the detection of SS, particularly in early-stage or seronegative patients.

## Introduction

Sjögren’s syndrome (SS) is a chronic autoimmune disorder primarily affecting the exocrine glands, leading to dryness of the mouth and eyes, and potentially involving systemic manifestations [1]. Accurate diagnosis is crucial for managing SS, given its varied clinical presentations and overlap with other autoimmune conditions like rheumatoid arthritis (RA) and systemic lupus erythematosus (SLE) [2-4]. The labial salivary gland biopsy (LSGB) is a key diagnostic tool for SS, providing histopathological evidence of lymphocytic infiltration that supports clinical findings [5,6]. However, its diagnostic accuracy, reliability, and role in conjunction with other diagnostic methods, such as serological testing, remain debated [7].

LSGB is often performed to assess the extent of lymphocytic infiltration in labial salivary glands, a hallmark of SS. The biopsy evaluates a "focus score," which quantifies the number of lymphocytic clusters within a 4 mm² area of glandular tissue. A score of ≥1 is typically considered indicative of SS [5]. LSGB offers an objective histopathological measure to support clinical suspicion and positive serological findings such as anti-Ro/SSA (anti-Ro/SS-A) and anti-La/SSB (anti-La/SS-B) antibodies. Studies have demonstrated its utility in confirming SS diagnoses, particularly in cases where serological markers are negative [6].

The sensitivity of LSGB for diagnosing SS varies among studies, ranging from 63% to 93%, depending on the criteria used and disease stage at biopsy [8]. LSGB tends to show higher sensitivity in patients with advanced diseases, where lymphocytic infiltration is more pronounced. In early-stage or seronegative patients, sensitivity may be lower due to minimal glandular inflammation. Specificity, however, remains high, consistently reported between 87% and 100%, indicating that LSGB is particularly effective at confirming SS when a positive result is obtained [9].

Although LSGB is a valuable tool, it is most effective when used in conjunction with other diagnostic modalities, such as serological testing and clinical evaluations. The presence of specific autoantibodies-anti-Ro/SSA (anti-Ro/SS-A) and anti-La/SSB (anti-La/SS-B)-provides an important serological indicator of SS, with approximately 60-70% of SS patients testing positive for these antibodies [10]. However, LSGB has particular value in seronegative patients, where a negative serological test result can complicate the diagnosis. Studies have demonstrated that LSGB improves diagnostic accuracy in these cases by providing objective histopathological confirmation [11,12].

In addition to the focus score, other histopathological features in LSGB, such as germinal center formation, acinar atrophy, and fibrosis, have been studied as additional markers of disease severity and progression. The presence of germinal centers is linked to a more aggressive disease course and a higher risk of lymphoma development in SS patients. Moreover, these features are particularly useful in differentiating between primary and secondary SS, which often presents with milder glandular involvement [13].

One of the primary challenges in using LSGB is the risk of false negatives, particularly in early-stage SS where lymphocytic infiltration is not yet substantial. Furthermore, the adequacy of the biopsy sample significantly impacts diagnostic accuracy, as inadequate samples can lead to inconclusive or false-negative results [14]. In addition, LSGB interpretation requires specialized pathology expertise to accurately quantify focus scores and recognize SS-specific histopathological patterns [15].

The diagnostic utility of LSGB differs between primary SS (pSS) and secondary SS (sSS). In pSS, where SS occurs in isolation, LSGB is generally more sensitive and specific due to more pronounced lymphocytic infiltration. By contrast, sSS, which coexists with other autoimmune diseases like RA or SLE, often shows less distinct glandular changes, complicating the biopsy’s interpretation. In these cases, the focus score might be lower, or overlapping autoimmune pathology may obscure the SS-specific findings [15].

LSGB has lower diagnostic accuracy in patients with early-stage SS or atypical presentations, where glandular involvement is minimal. Atypical symptoms such as fatigue or neurological manifestations, without prominent sicca symptoms, may lead to lower lymphocytic infiltration in the salivary glands, resulting in false negatives, highlighting the importance of combining LSGB with other diagnostic tools, particularly serological and clinical assessments, to avoid misdiagnosis in these cases [16].

Despite its utility, LSGB has several limitations. The invasive nature of the procedure can cause patient discomfort, and complications such as pain, swelling, or infection have been reported, although these are generally mild and self-limiting. Moreover, the quality of the biopsy sample plays a critical role in the accuracy of the results. If the biopsy sample is too small or poorly preserved, it may not provide enough tissue for an accurate diagnosis, increasing the risk of false negatives [8].

From a cost-effectiveness perspective, LSGB is relatively inexpensive compared to advanced imaging techniques or extensive serological testing. It offers the potential to provide a definitive diagnosis, thus avoiding unnecessary further investigations. However, the need for repeat biopsies in cases of inadequate samples or inconclusive results can increase costs and reduce overall cost-efficiency.

LSGB remains a crucial diagnostic tool for SS, particularly in cases where serological markers are absent or inconclusive. It offers high specificity and moderate sensitivity, making it a reliable method for confirming SS when positive. However, its diagnostic accuracy can be affected by sample adequacy, disease stage, and coexisting autoimmune conditions, particularly in secondary SS. LSGB should be used in conjunction with other diagnostic methods to improve accuracy, especially in early-stage or atypical presentations of SS. Further research is required to refine the use of LSGB and explore its role in early diagnosis and clinical decision-making.

## Materials and methods

The primary aim of this study is to evaluate the reliability of the LSGB as a diagnostic tool for SS.

Study settings and design

The study constituted a retrospective analysis of patients treated at the Department of Ear, Nose, and Throat (ENT) at Salmaniya Medical Complex (SMC), a facility within the government hospitals (GHs) in the Kingdom of Bahrain. It utilized previously collected data from patient records, encompassing pathology reports and follow-up clinical visits, while ensuring that no direct patient interactions or additional interventions occurred. Records were extracted from the information system (iSeha), a comprehensive electronic medical records platform employed across GHs in the Kingdom of Bahrain, which integrated patient data systematically.

Study duration

The seven-year study (2016-2023) encompassed a diverse patient sample, thereby enhancing generalizability. It coincided with the implementation of a standardized pathology reporting system, which ensured consistent biopsy evaluations throughout the study period.

Study population and sample size

The study population comprised patients who underwent LSGB between 2016 and 2023 for the evaluation of SS. The sample included all eligible patients identified from the medical records who met the established inclusion and exclusion criteria. The anticipated sample size was estimated to range from 200 to 300 patients, although the exact number would be determined upon final data extraction. This sample size was deemed adequate to assess the diagnostic accuracy of LSGB across various subgroups, including those with primary and secondary SS, as well as patients exhibiting different clinical symptoms.

Inclusion Criteria

Patients included in the study met the following criteria: they underwent LSGB to investigate SS; had complete pathology reports, clinical data, and laboratory findings relevant to SS in their medical records; and received a specialist diagnosis of SS from a rheumatologist, regardless of whether the diagnosis was confirmed or ruled out based on LSGB results.

Exclusion Criteria

Patients were excluded from the study if their clinical data was incomplete, particularly if insufficient documentation existed to confirm or refute an SS diagnosis. In addition, patients who underwent LSGB before September 2016, when the new pathology reporting system was implemented, were excluded. 

Data collection

Data collection was conducted through iSeha, the GH's electronic health record system, to gather comprehensive patient information, including demographics, clinical symptoms, laboratory findings, LSGB results, and specialist diagnoses. Key data parameters included age, gender, ethnicity, and duration of symptoms. Clinical symptoms such as dry eyes, dry mouth, fatigue, and neurological issues were documented. Laboratory findings, including antinuclear antibody (ANA) titers, rheumatoid factor (RF), and anti-Ro/SSA and anti-La/SSB antibodies, were analyzed. LSGB results focused on the focus score, sample adequacy, and histopathological features. Finally, rheumatologist-confirmed diagnoses of SS enabled comparison with biopsy results to assess the diagnostic accuracy of LSGB.

Data management and confidentiality

Data were anonymized, assigned unique identifiers, and securely stored, with access restricted to authorized personnel. The comparison was tested using the Chi-square test for all categorical data, and the t-test was used for continuous data. Statistical analysis focused on evaluating the diagnostic reliability of LSGB in SS through sensitivity, specificity, positive predictive value (PPV), and negative predictive value (NPV). Descriptive statistics presented continuous variables as mean ± standard deviation or median and interquartile range, while categorical variables were reported as frequencies. Diagnostic accuracy was measured using a 2 x 2 contingency table and Cohen’s kappa coefficient. Subgroup analyses assessed primary versus secondary SS, and a cost-effectiveness analysis evaluated the efficiency of LSGB in conjunction with serological tests.

Ethical considerations

This study adhered to the ethical principles outlined in the Declaration of Helsinki and conformed to the four primary ethical principles of autonomy, beneficence, non-maleficence, and justice. As a retrospective study, no new interventions were performed, and patient data were managed in a manner that respected patient autonomy and confidentiality. All patients included in the study had previously consented to the use of their data for research purposes upon their admission to the hospital. The study received ethical approval from the Ethical Committee of Government Hospitals (research approval serial no.: 53-290424).

## Results

The key findings from the analysis of LSGB outcomes in patients suspected of having SS explore associations between LSGB results and various clinical, demographic, and serological factors.

The characteristics of the study population (n = 166) are captured in Table 1. The mean age of the participants was 45.2 ± 14 years, indicating that the majority of participants were middle-aged. The gender distribution showed a notable predominance of female participants, with 148 (89.1%) females compared to 18 (10.8%) males. This imbalance reflects the epidemiology of many autoimmune diseases, where women are affected at a much higher rate than men.

**Table 1:** Demographic variables

Variables	N = 166
Age in yrs. Mean (SD)	45.2 (± 14.0)
Gender, N (%)	
Male	18 (10.8%)
Female	148 (89.1%)
Age group, N (%)	
Pediatric	10 (6.0%)
Adult	156 (93.9%)

Further stratification by age group portrayed that the vast majority of participants were adults, with 156 (93.9%) adults, while pediatric participants accounted for only a small proportion of the sample, with 10 (6.0%) pediatric participants, showcasing SS primarily as an adult-onset or adult-prevalent disorder, with limited pediatric involvement.

The relationship between various laboratory findings and the results of LSGB in a cohort of patients suspected of having SS, including ANA, RF, and the specific SS-related antibodies (anti-Ro and anti-La), were categorized as either "suggestive" or "non-suggestive" of SS, and the statistical significance of the associations between these antibodies and biopsy outcomes was evaluated using P-values (Table 2).

**Table 2:** Laboratory findings vs. labial salivary gland biopsy LSGB: labial salivary gland biopsy, ANA: antinuclear antibodies, RF: rheumatoid factor, anti-Ro: anti-Ro (SS-A) antibodies, anti-La: anti-La (SS-B) antibodies

Marker	Suggestive LSGB results (N,%)	Non-suggestive LSGB results (N,%)	P-value
ANA			
Positive	38 (45%)	46 (54%)	0.003
Negative	17 (22%)	57 (78%)	
RF			
Positive	7 (36%)	12 (64%)	0.394
Negative	30 (27%)	80 (73%)	
Anti Ro			
Positive	40 (54%)	33 (46%)	0.062
Negative	18 (37%)	30 (62%)	
Anti La			
Positive	32 (40%)	47 (60%)	0.669
Negative	20 (44%)	25 (56%)	

Just 17 (22%) of the ANA-negative patients showed suggestive findings, compared to 38 (45%) of ANA-positive patients who had suggestive LSGB results. However, while 57 (78%) of ANA-negative patients exhibited non-suggestive LSGB findings, 45 (54%) of the ANA-positive patients had non-suggestive results. ANA positivity and suggestive LSGB results were found to be significantly correlated (P = 0.003), enabling researchers to consider ANA as a helpful predictor of histological evidence of SS. Although ANA is commonly acknowledged as a nonspecific indicator of autoimmunity, its high frequency in SS makes it a crucial diagnostic component, particularly when paired with histological evidence and clinical symptoms.

By contrast, there was no significant correlation between RF and LSGB results (P = 0.394). Compared to 30 (27%) of RF-negative individuals, seven (36%) of RF-positive patients exhibited suggestive LSGB results. Similarly, 80 (73%) of RF-negative individuals had non-suggestive LSGB results, while 12 (64%) of RF-positive patients did. RF was found to have poor diagnostic usefulness in SS, especially when compared to other biomarkers like ANA and anti-Ro antibodies, even though it is frequently high in autoimmune illnesses like RA.

With a p-value of 0.062, the association between anti-Ro antibodies and LSGB outcomes was close to statistical significance. Compared to 18 (37%) anti-Ro-negative patients, 40 (54%) patients with positive anti-Ro antibodies exhibited suggestive LSGB results. Conversely, 30 (62%) of anti-Ro-negative patients had non-suggestive LSGB results, while 33 (46%) of anti-Ro-positive patients did. This pattern implies that anti-Ro antibodies might be linked to suggestive LSGB findings, even though they did not meet the statistical significance level. When patients have seronegative results for other markers, anti-Ro, a well-known biomarker for SS, is part of the diagnostic criteria. Although more research with larger sample sizes may be necessary to validate this relationship, the near-significant association in this cohort raises the possibility that anti-Ro antibodies may have predictive utility for LSGB outcomes.

Twenty (44%) patients with anti-La-negative results exhibited suggestive LSGB findings, compared to 32 (40%) individuals with positive anti-La antibodies. Similarly, 25 (56%) anti-La-negative individuals showed non-suggestive LSGB results, while 47 (60%) anti-La-positive patients had non-suggestive results. Despite being crucial in the serological diagnosis of SS, anti-La antibodies may not be a reliable indicator of histological evidence from LSGB, according to this lack of significant correlation. Although anti-La and anti-Ro antibodies are frequently found together in SS patients, their diagnostic utility seems to be restricted in this study population. No significant link between the presence of anti-La antibodies and LSGB outcomes was observed (P = 0.669).

**Figure 1:**
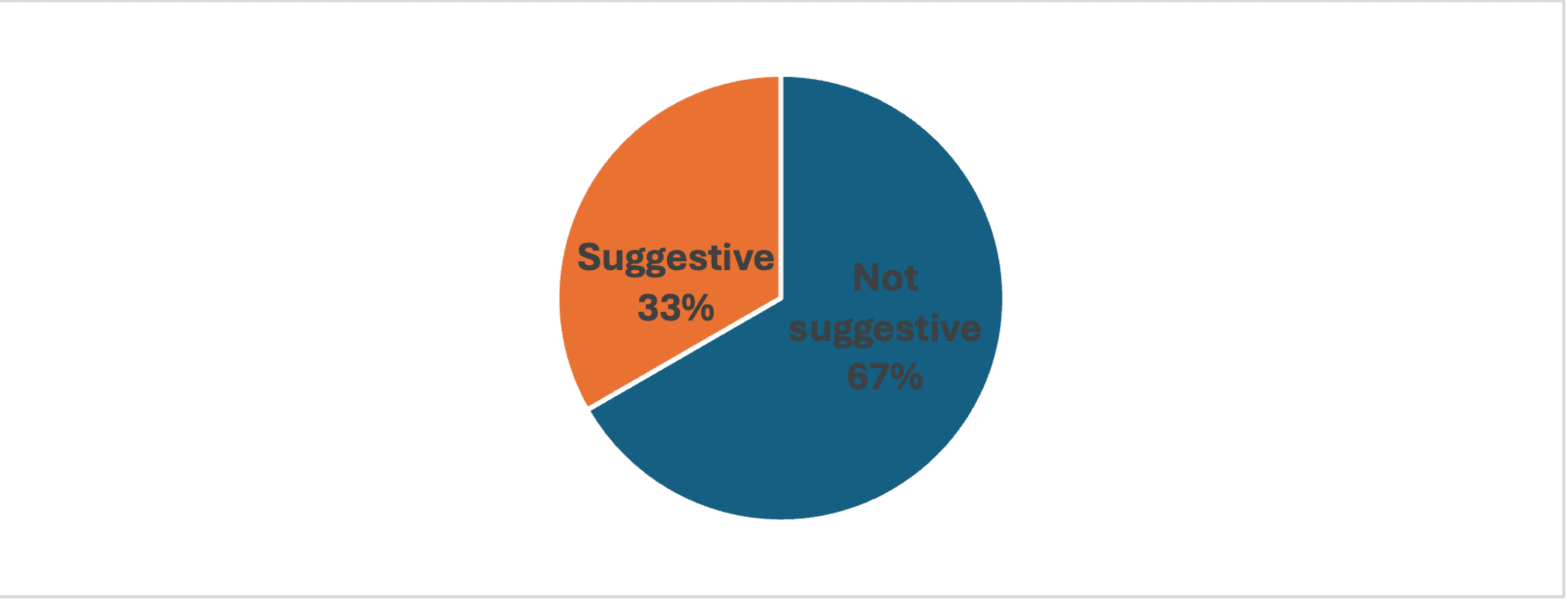
Labial salivary gland biopsy result: suggestive/not suggestive

A third of the LSGB results were "suggestive" of SS (55 patients, 33%), showing supportive histological evidence. By contrast, two-thirds (111 patients, 67%) of the LSGB results were deemed "not suggestive," depicting a lack of adequate diagnostic criteria. This highlights the prerogative to combine LSGB with clinical and serological examinations to arrive at the correct diagnosis, as the majority of patients did not exhibit definitive biopsy results (Figure 1).

The relationship between the results of LSGB and the specialist diagnosis of SS (Table 3) illustrated that of the 166 patients, 111 (66.9%) had LSGB that is not suggestive of SS or had passed away, whereas 55 (33.1%) had LSGB suggestive results. Of the diagnosed patients, 22 (40%) had "not suggestive" results, and 33 (60%) had LSGB results suggestive of SS, implicating that LSGB findings may be erratic and do not always coincide with the clinical evaluation of the professional.

**Table 3:** Specialist diagnosis LSGB: labial salivary gland biopsy

Specialist diagnosis	LSGB not suggestive (N,%)	LSGB suggestive (N,%)	Total	P-value
Diagnosed	22 (40%)	33 (60%)	55	< 0.001
Not diagnosed	88 (80%)	22 (20%)	110	
Patient died	1 (100%)	0 (0%)	1	
Total	111 (66.9%)	55 (33.1%)	166	

In a similar vein, among the 110 patients without an SS diagnosis, 22 (20%) showed suggestive LSGB results, while 88 (80%) had "not suggestive" results. The cohort included one patient who passed away, and their LSGB results were deemed "not suggestive." The data point about this patient was probably added for completeness's sake, but it has no discernible impact on the analysis.

The study, thus, suggests a very strong correlation between the biopsy findings and a specialist's clinical diagnosis of SS (P < 0.001) and emphasizes that LSGB by itself is not always conclusive because biopsy results can differ from the clinical diagnosis in certain situations. This substantiates how crucial it is to use a multimodal strategy to diagnose SS, combining histopathological, clinical, and serological data to increase diagnostic precision.

The relationship between the age of the patient and LSGB outcomes reflected that those with "not suggestive" biopsy results (n = 111) had a mean age of 44.85 years (SD = 15.00), while patients with "suggestive" results (n = 55) had a slightly higher mean age of 46.14 years (SD = 12.32), denoting that age did not influence in the LSGB results (P=0.580) (Table 4).

**Table 4:** Age vs. labial salivary gland biopsy results

	LSGB result	Number	Mean	Std. deviation	P-value
Age	Not suggestive	111	44.85	15.00	0.580
	Suggestive	55	46.14	12.32	

Schirmer's test, a diagnostic procedure used to assess tear production, can determine if a patient has dry eyes, a common symptom of conditions like SS. The test involves a small strip of filter paper placed inside the lower eyelid for a few minutes to measure tear production. The result is typically normal when there is wetting of 10 to 15 millimeters of the paper after five minutes. Reduced tear production, indicated by less wetting of the paper, may suggest dry eye disease or other disorders affecting the lacrimal glands, such as autoimmune conditions like SS. The test was performed by the Ophthalmology department, and the results were noted.

An analysis (Table 5) of Schirmer's test results and LSGB in the cohort was done to understand the correlation between the lab and biopsy findings. Among the three patients who had abnormal test results, the LSGB findings of only one (33.3%) were "suggestive," while the other two (66.6%) were “not suggestive,” indicating that the biopsy did not detect SS despite abnormal Schirmer's test findings. This suggests that the sensitivity of the LSGB in this small subset is relatively low, as it failed to detect SS in patients who likely had the condition based on abnormal test results.

**Table 5:** Schirmer test vs. labial salivary gland biopsy result LSGB: labial salivary gland biopsy

Test	LSBG not suggestive (N, %)	LSGB suggestive (N, %)	Total	P-value
Done: abnormal	2 (66.6%)	1 (33%)	3	0.980
Done: normal	9 (81.8%)	2 (18.2%)	11	
Not done	39 (60%)	26 (40%)	65	
Not referred	61 (70.1%)	26 (29.9%)	87	
Total	111 (66.9%)	55 (33.1%)	166	

Conversely, in the group with normal test results, two (18.2%) patients had "suggestive" LSGB findings, suggesting good specificity but also highlighting the occurrence of probable false positives. LSGB indicated SS, despite Schirmer's test results being normal. The remaining nine (81.8%) patients had "not suggestive" LSGB findings, representing true negatives, where both the test and biopsy aligned in ruling out SS.

A larger third of patients did not undergo the test (n = 65), although they were referred to the Ophthalmology department. Of these patients, 39 (60%) had "not suggestive" LSGB results, and 26 (40%) had "suggestive" results. Of the 87 patients who were "not referred" to the Ophthalmology department, from either the ENT or Dermatology departments, 61 (70.1%) patients had "not suggestive" biopsy results, while 26 (29.9%) had "suggestive" results, further supporting the assumption that LSGB can provide valuable diagnostic information independent of other tests.

Thus, LSGB outcomes may be considered independent of whether the test was performed, its results were abnormal or normal, or the patient was referred for the test (P = 0.980), depicting the relative robustness of LSGB as a diagnostic tool for SS.

While considering other autoimmune diseases and their influence on LSGB results, 86 (72.9%) had "not suggestive" LSGB results, while only 32 (27.1%) had "suggestive" results of the 118 patients without other autoimmune diseases, leading to the notion that most patients without other autoimmune diseases did not exhibit histopathological signs of SS (Table 6).

**Table 6:** Other autoimmune diseases LSGB: labial salivary gland biopsy

Other autoimmune diseases	LSGB not suggestive (N,%)	LSGB suggestive (N,%)	Total	P-value
None	86 (72.9%)	32 (27.1%)	118	0.034
Yes	25 (52.1%)	23 (47.9%)	48	
Total	111 (66.9%)	55 (33.1%)	166	

By contrast, among the 48 patients with other autoimmune diseases, 25 (52.1%) had "not suggestive" LSGB results, and 23 (47.9%) had "suggestive" results. The higher proportion of suggestive LSGB results in patients with other autoimmune diseases may reflect the complex interplay of additional autoimmune conditions, such as RA or lupus, which are more likely to have biopsy findings suggesting SS.

The significant p-value of 0.034 highlights the need for a comprehensive approach to diagnosing SS, considering coexisting autoimmune diseases when interpreting LSGB results, as patients with multiple autoimmune conditions may exhibit heightened lymphocytic infiltration in their salivary glands. Thus, we must take into account not only the biopsy results but also the broader clinical context, especially in patients with additional health conditions.

## Discussion

LSGB remains a crucial diagnostic tool in clinical practice, particularly in ambiguous or seronegative cases of SS. The primary objective of this study is to assess the diagnostic accuracy of LSGB in identifying SS, with a specific focus on correlating biopsy results with clinical, serological, and demographic factors.

Research has previously demonstrated LSGB’s high specificity, particularly when used in combination with clinical and serological assessments [15,17,18]. With the present study yielding a third of the biopsies (33%) being suggestive of SS, it corroborates LSGB’s sensitivity values ranging from 63% to 93% and a specificity of 87% to 100%, depending on disease stage and biopsy technique [19]. Sixty-seven percent of the biopsies in our study were deemed non-suggestive, highlighting the possibility of misleading negative results, particularly in patients with moderate or early illness. This is consistent with research by Jonsson et al. (2010), who found that in the early stages of SS, when glandular involvement is less noticeable, LSGB sensitivity declines [20].

ANA has been shown to be a useful marker in SS diagnosis, particularly when combined with LSGB [13,21]. In Manoussakis’ cohort, ANA-positive patients had a higher likelihood of exhibiting suggestive biopsy findings, which aligns with our results, where ANA positivity was significantly associated with suggestive LSGB outcomes [13].

Brito-Zerón et al. (2018) reported that RF is less specific for SS compared to ANA or Anti-Ro antibodies, similar to the present research [8]. Anti-La, often present alongside Anti-Ro in SS, also did not show a significant correlation with biopsy findings. However, anti-La’s diagnostic value is more limited when compared to other serological markers like anti-Ro [21,22]. By contrast, the trend toward significance with anti-Ro in our study (p = 0.062) suggests that this marker remains a strong predictor of suggestive LSGB findings. Anti-Ro antibodies are recognized as highly specific for SS and have been included in multiple diagnostic criteria, including the 2016 ACR-EULAR criteria [23].

The limitations of LSGB as a standalone diagnostic tool are highlighted by the 67% non-suggestive biopsies in our cohort, emphasizing the need for additional diagnostic modalities. Brito-Zerón et al. (2018) discussed the role of salivary gland ultrasonography (SGUS) as a non-invasive, complementary tool to LSGB, showing that SGUS can detect glandular changes indicative of SS, even in cases where LSGB fails to provide conclusive results [24]. SGUS, while not as specific as LSGB, has been shown to have a sensitivity ranging from 61% to 91%, depending on the disease stage, making it a valuable addition to the diagnostic workup in SS [25]. Our findings align with the growing consensus that LSGB, while specific, can miss early or subtle cases of SS and should be supported by imaging or additional serological tests.

The significant association between other autoimmune diseases and suggestive LSGB results (p = 0.034) in the study suggests that patients with other autoimmune conditions or systemic illnesses are more likely to exhibit histopathological changes consistent with SS. Patients with RA or systemic lupus erythematosus (SLE) often display overlapping histological features in the salivary glands [26]. Diagnosing SS in patients with secondary SS, where LSGB may yield false-positive results due to glandular involvement from the primary autoimmune disease, remains challenging, necessitating clinicians to rely on the entire clinical picture and use LSGB results with caution, as the histological changes may not be specific to SS [6,27,28]. Patients with multiple autoimmune conditions often display increased lymphocytic infiltration, complicating the differentiation between primary and secondary SS [29].

While SS predominantly affects middle-aged women, our data suggest that LSGB can be used effectively in both younger and older populations without age being a significant confounding factor. LSGB remains a reliable diagnostic tool across a wide age range, from younger adults to older individuals [30,31]. Comorbid conditions, such as RA and SLE, can complicate the interpretation of LSGB results, leading to a higher rate of false positives.

Another important diagnostic tool is Schirmer's test. While the test may indicate ocular dryness, it is not always aligned with histopathological findings of SS, echoing research by Shiboski et al. (2017), which emphasizes the variable nature of SS presentations [23]. It has been seen that Schirmer's test alone has limited specificity and should not be relied upon independently for SS diagnosis [31]. Again, such findings emphasize the importance of a multi-modal approach, as individual tests may not capture the full disease spectrum.

Limitations

The limitations of the study need to be acknowledged despite its value. False-negative results, particularly in early-stage disease, remain a significant challenge. In our study, the high percentage of non-suggestive LSGB results (67%) underscores the potential for missing early or subtle cases of SS. The sample size, although reasonable, may limit the generalizability of the findings to larger, more diverse populations. The patient cohort drawn from a specific geographic region could introduce regional biases related to disease prevalence, access to healthcare, or diagnostic practices. Moreover, the retrospective nature of the study may lead to inherent biases in data collection, particularly in terms of missing or incomplete records.

In addition, while LSGB is a valuable diagnostic tool, it is not without variability, especially regarding sample adequacy and pathologist interpretation, which could have influenced the results. False negatives may occur due to insufficient biopsy material or early-stage disease, and false positives might arise in cases with overlapping histopathological features from other conditions. While LSGB remains the gold standard, these non-invasive imaging techniques offer promising complementary options, particularly for patients who may be at risk of false-negative results on biopsy.

## Conclusions

The valuable role of LSGB in diagnosing SS, particularly when integrated with clinical and serological findings, is the focus of this study. LSGB proved to be a valuable diagnostic tool, with 33% of biopsies yielding suggestive results for SS. However, it is not definitive on its own. Schirmer's test, while useful for detecting ocular dryness, demonstrated limited correlation with LSGB findings, reinforcing the need for additional diagnostic tools beyond individual tests. Notably, the presence of other autoimmune diseases significantly increases the likelihood of suggestive LSGB findings, emphasizing the need for careful consideration of patient health profiles during diagnosis, while, in contrast, age did not affect it. Significant ANA positivity reinforces the importance of serological markers in the diagnostic process.

The study thus substantiates the importance of a comprehensive, multi-modal diagnostic approach, combining LSGB, clinical evaluation, and laboratory markers to improve diagnostic accuracy and patient outcomes in SS. LSGB should continue to be used as part of the diagnostic workup for SS, particularly in patients with inconclusive serological findings or where clinical symptoms are ambiguous. However, clinicians should be aware of the limitations of LSGB, particularly the risk of false negatives in early-stage disease or mild glandular involvement.

To address the limitations, future research should consider a larger, multi-center study with a more diverse population to improve the generalizability of the findings. Prospective studies would also allow for more controlled data collection and a deeper exploration of how LSGB findings correlate with disease progression and patient outcomes over time.

Further studies should examine additional biomarkers and advanced imaging techniques, such as salivary gland ultrasonography, to determine their potential as complementary diagnostic tools to LSGB. In addition, future research should explore the specific impact of individual comorbid conditions on LSGB results to provide a clearer understanding of how coexisting diseases influence the histopathological findings in SS.
